# Impacts of Dynamic Agglomeration Externalities on Eco-Efficiency: Empirical Evidence from China

**DOI:** 10.3390/ijerph15102304

**Published:** 2018-10-19

**Authors:** Yantuan Yu, Yun Zhang, Xiao Miao

**Affiliations:** 1 School of Economics and Trade, Hunan University, Changsha 410079, China; yantuanyu@hnu.edu.cn; 2 School of Finance, Shanghai Lixin University of Accounting and Finance, Shanghai 201620, China; 3 Innovation Research Institute of Traditional Chinese Medicine, Shanghai University of Traditional Chinese Medicine, Shanghai 201203, China

**Keywords:** dynamic agglomeration externalities, eco-efficiency, nonconvex metafrontier, super-efficiency, slacks-based measure

## Abstract

Ecological efficiency (eco-efficiency) reflects the synergetic degree of the development of resource, economic, and environmental systems. This paper measures urban eco-efficiency based on a nonconvex metafrontier data envelopment analysis (DEA) approach using data from 191 cities in China during the years of 2003 to 2013. In particular, the impacts of dynamic agglomeration externalities on urban eco-efficiency are investigated. Our empirical results show that eco-efficiency decreased from 2003 to 2013, and its spatial distribution demonstrates significant regional heterogeneity. Additionally, there exists an inverted U-shape relationship between dynamic externalities, including Marshall-Arrow-Romer (MAR), Jacobs and Porter externalities, and eco-efficiency. We also find that eco-efficiency can be enhanced by strengthening environmental regulations, optimizing industrial structures, and improving technological capacity. These findings are robust to alternative eco-efficiency measures, model specifications, and estimation approaches. Furthermore, we discuss related policy implications of our research results.

## 1. Introduction

In this paper, we investigate how dynamic agglomeration externalities of Chinese prefecture-level cities relate to ecological efficiency (henceforth eco-efficiency). More specifically, we examine the nonlinear effects that Marshall-Arrow-Romer (MAR), Jacobs, and Porter externalities exert on eco-efficiency. Generally, the formation of industrial clusters can improve resources utilization efficiency, positively impacting eco-efficiency. Yet, industrial over-agglomeration may have negative influences on eco-efficiency because of the potential severe environmental pollution. Thus, the relationship between dynamic agglomeration externalities and eco-efficiency may show significant inverted U-shape form. To test this hypothesis, we empirically study the nonlinear effects of dynamic agglomeration externalities on China’s eco-efficiency using data from 191 cities during the years of 2003 to 2013.

Extensive studies have been devoted to defining eco-efficiency. In order to provide a comprehensive evaluation of both the environmental and the economic performance of firms, the World Business Council for Sustainable Development (WBCSD) [1] proposed the concept of eco-efficiency, one of the main tools to promote a transformation from unsustainable development to one of sustainable development. It is based on the concept of creating more goods and services while using fewer resources and creating less waste and pollution: “It is measured as the ratio between the (added) value of what has been produced (e.g., GDP) and the (added) environment impacts of the product or service (e.g., CO_2_ emissions)”. Varied definitions of eco-efficiency also defined by other international organizations such as the Organization for Economic Cooperation and Development (OECD) [2] and European Environment Agency (EEA) [3]. Basically, the underlying idea behind the concepts of eco-efficiency is that it is a good thing to produce more desirable output with less undesirable output, which also reflects the sustainable development of resource, economic, and environmental systems. Generally, it can be argued that the coordinated promotion of eco-efficiency among cities is one of the primary ways to implement sustainable development in the presence of resource exhaustion and environmental pollution.

There has been a growing literature on measuring the eco-efficiency using parametric [4,5,6] or nonparametric approaches [7,8,9,10,11,12,13,14,15,16,17]. Parametric and nonparametric approaches differ primarily in the assumptions they use when estimating the efficient frontiers. Stochastic frontier analysis (SFA) and data envelopment analysis (DEA) are the most employed parametric and nonparametric methods in the literature, respectively. The SFA assumes composed errors that distinguish statistical noise from the technical inefficiency term, while it requires specifying a particular functional form, which determines the shape of efficient frontier. However, we do not know which prespecified functional form is consistent with the data, e.g., Cobb–Douglas and translog specifications, estimation results of these two functions maybe various and it is difficulty to decide which one performs better. Consequently, it may lead to biased results for the selected functional form introduces inductive bias in the stochastic analysis. More importantly, it is also hard to deal with multiple outputs especially the undesirable outputs [18]. Conversely, the DEA has several strengths such as being free from specifying a functional form and it can deal with multiple inputs and multiple outputs. Its direct, data-driven approach is helpful for communicating the results of efficiency analysis to decision-makers. Several scholars incorporate metafrontier technique into either stochastic frontier analysis (SFA) or data envelopment analysis (DEA) to investigate heterogeneity factors [19,20]. However, previous studies mainly focus on the convex metafrontier, which may lead to biased estimation of eco-efficiency since the convex metafrontier contains the “infeasible input–output combinations” [21,22,23,24]. To rank and differentiate efficient decision-making units (DMUs), a super-efficiency model has be proposed by [25] so that efficient DMUs can have efficiency scores greater than unity and the efficiency scores of the inefficient DMUs are remain unchanged. Researchers adopt nonconvex metafrontier method to analyze efficiency and productivity, however, the super-efficiency model [25] has not been fully considered in measuring efficiency and the efficient DMUs cannot be further rank. Thus, in this paper, one of our objectives is to measure eco-efficiency based on an extended DEA model incorporating both nonconvex metafrontier and super-efficiency into the slacks-based measure [26]. Furthermore, we investigate how agglomeration externalities of Chinese prefecture-level cities relate to eco-efficiency.

Agglomeration economies or external economies of scale refer to the benefits from concentrating output and housing in particular areas. The fundamental idea rests upon the advantages accruing both on the demand and the supply side to firms located geographically close to each other. However, as emphasized previously [27], agglomeration can also bring negative effects by producing congestion costs, which are also known as the negative externalities of agglomeration. Agglomeration externalities have attracted considerable attention from researchers. There has been a rich empirical literature on agglomeration externalities [28], such as the meta-analysis of agglomeration economies provided previously [29]. Specifically, several empirical studies focus on agglomeration and economic growth [30], agglomeration and environmental performance [31], agglomeration and firm productivity [32], and agglomeration and energy efficiency [33,34]. While the results from the above-mentioned studies have important implications on the relationship between agglomeration externalities and eco-efficiency, the latter is an aggregation indicator simultaneously considering three fundamental perspectives: economic growth, environmental pollution, and resources utilization. To the best of our knowledge, there has been no empirical study to examine the impacts of agglomeration externalities on eco-efficiency. In this paper, we try our best to fill this research gap.

The last few years have witnessed a growing literature on the relationship between agglomeration and efficiency or productivity. For example, the eco-efficiency increased by 30 to 40% due to industrial clusters [35]. The relationship between manufacturing agglomeration and environmental technological efficiency forms an inverted U-shaped curve [36]. However, the Marshall-Jacobs’s self-cleaning effect leads to a U-shaped relationship between industrial agglomeration and environmental efficiency [37]. Recently, the authors of a previous paper [38] showed that the impact of industrial agglomeration on energy efficiency has a threshold effect in the eastern region of China. Similarly, only when agglomeration reaches a certain level will industrial agglomeration positively impact on industrial energy efficiency improvement [33]. 

The rest of the paper is organized as follows: the methods, variables and data are explained in the next section, followed by the empirical results in Section 3. In Section 4, conclusions and further research are presented.

## 2. Methods, Variables and Data

### 2.1. Empirical Models

To investigate the nonlinear effects of dynamic agglomeration externalities on urban eco-efficiency, we introduce the quadratic term of dynamic agglomeration externalities into the empirical model, which is specified as

(1) $$\;ee_{it}=c+\beta_{0}ext_{it}+\beta_{1}ext_{it}^{2}+X\xi +\mu_{i}+\tau_{t}+u_{it}\;$$

where $ee_{it}$ is the eco-efficiency of city i in period t, $ext_{it}$ indicates the agglomeration dynamic externalities of city i in period t, $ext_{it}^{2}$ is the quadratic term of $ext_{it}$, X denotes control variables, $\mu_{i}$ and $\tau_{t}$ represent the individual fixed and time fixed effects, and $u_{it}$ captures the stochastic perturbation. $\beta_{0}$, $\beta_{1}$, and ξ are the coefficients needed to be estimated.

We also study the dynamic effects of eco-efficiency by introducing its lagged term into Equation (1), which is estimated as

(2) $$\;ee_{it}=c+\alpha ee_{it-1}+\beta_{0}ext_{it}+\beta_{1}ext_{it}^{2}+X\xi +\mu_{i}+\tau_{t}+u_{it}\;$$

where $ee_{it-1}$ denotes the lagged term of eco-efficiency of city i in period t and α represents the impact of $ee_{it-1}$ on $ee_{it}$.

Finally, we examine the spatial effects of three types externalities on eco-efficiency. In this study, we construct two types of spatial weights matrices, i.e., inverse distance (ID) matrix and economic-distance-based (ED) matrix. The ED matrix is used as a robustness test and the ID matrix specifies the inverse geographical distance between each two spatial units:

(3) $$\;w_{ij}=\{\begin{array}{cc} 0, & if\;i=j\\ \frac{1/d_{ij}}{\sum_{j}1/d_{ij}}, & otherwise \end{array}$$

where $d_{ij}$ represents the geographic distance between cities i and j (unit: km).

Spatial model specifications are specified as follows [39].

SAR model:

(4) $$\;ee_{it}=c+\beta_{0}ext_{it}+\beta_{1}ext_{it}^{2}+\rho Wee_{it}+X\xi +\mu_{i}+\tau_{t}+u_{it}\;$$

SEM model:

(5) $$\;ee_{it}=c+\beta_{0}ext_{it}+\beta_{1}ext_{it}^{2}+X\xi +\mu_{i}+\tau_{t}+u_{it};u_{it}=\lambda Wu_{it}+\varepsilon_{it}\;$$

SDM model:

(6) $$\;ee_{it}=c+\beta_{0}ext_{it}+\beta_{1}ext_{it}^{2}+\rho Wee_{it}+X\xi +WX\theta +\mu_{i}+\tau_{t}+u_{it}\;$$

SAC model:

(7) $$\;ee_{it}=c+\beta_{0}ext_{it}+\beta_{1}ext_{it}^{2}+\rho Wee_{it}+X\xi +WX\theta +\mu_{i}+\tau_{t}+u_{it};u_{it}=\lambda Wu_{it}+\varepsilon_{it}\;$$

where W is the spatial weight matrix, ρ is the spatial autoregressive coefficient, whose absolute value is smaller than 1.

### 2.2. Variables Specification

#### 2.2.1. Dependent Variable

The dependent variable in our empirical models is urban eco-efficiency ($ee_{it}$), measured by the proposed DEA model, which is illustrated in detail as follows. Assuming that the number of DMUs is N, according to their heterogeneity, they can be divided into G (G>1) groups, each group containing $N_{g}$ DMUs. Then we have $\sum_{g=1}^{G}N_{g}=N$. There are three types of factors for each DMU: input variables, desirable outputs, and undesired outputs, expressed as the following. $x=\left[x_{1},x_{2},\cdots ,x_{M}\right]\in {\mathbb{R}}_{+}^{M}$, $y=\left[y_{1},y_{2},\cdots ,y_{R}\right]\in {\mathbb{R}}_{+}^{R}$, and $b=\left[b_{1},b_{2},\cdots ,b_{R}\right]\in {\mathbb{R}}_{+}^{J}$, respectively. M, R, and J represent the number of the three types of variables. Considering the super-efficiency and the metafrontier, the convex production possible set $P^{c-meta}$ and the nonconvex production possible set $P^{nc-meta}$ of the first decision unit in the first group are, respectively, defined as

$$\;P^{c-meta}=\{\left(x_{m},y_{r},b_{j}\right)|x_{mg^{\prime }o}\geq \overset{G}{\underset{g=1}{\sum }}\overset{}{\underset{n\in g,n\neq o\;if\;g=g^{\prime }}{\sum }}\varphi_{gn}x_{mgn},\;m=1,2,\cdots ,M;\;$$

$$\;y_{rg^{\prime }o}\leq \overset{G}{\underset{g=1}{\sum }}\overset{}{\underset{n\in g,n\neq o\;if\;g=g^{\prime }}{\sum }}\varphi_{gn}y_{rgn},\;r=1,2,\cdots ,R;\;$$

$$\;b_{jg^{\prime }o}\geq \overset{G}{\underset{g=1}{\sum }}\overset{}{\underset{n\in g,n\neq o\;if\;g=g^{\prime }}{\sum }}\varphi_{gn}b_{jgn},\;j=1,2,\cdots ,J;\;$$

$$\;\overset{G}{\underset{g=1}{\sum }}\overset{}{\underset{n\in g,n\neq o\;if\;g=g^{\prime }}{\sum }}\varphi_{gn}=1;\;\varphi_{gn}\geq 0;\;$$

(8) $$\;g=1,2,\cdots ,G;\;n\in g,n\neq o\;if\;g=g^{\prime }.\}\;$$

$$\;P^{nc-meta}=\{\left(x_{m},y_{r},b_{j}\right)|x_{mg^{\prime }o}\geq \overset{G}{\underset{g=1}{\sum }}\overset{}{\underset{n\in g,n\neq o\;if\;g=g^{\prime }}{\sum }}\gamma_{gn}x_{mgn},\;m=1,2,\cdots ,M;\;$$

$$\;y_{rg^{\prime }o}\leq \overset{G}{\underset{g=1}{\sum }}\overset{}{\underset{n\in g,n\neq o\;if\;g=g^{\prime }}{\sum }}\gamma_{gn}y_{rgn},\;r=1,2,\cdots ,R;\;$$

$$\;b_{jg^{\prime }o}\geq \overset{G}{\underset{g=1}{\sum }}\overset{}{\underset{n\in g,n\neq o\;if\;g=g^{\prime }}{\sum }}\gamma_{gn}b_{jgn},\;j=1,2,\cdots ,J;\;$$

$$\;\overset{G}{\underset{g=1}{\sum }}\overset{}{\underset{n\in g,n\neq o\;if\;g=g^{\prime }}{\sum }}\gamma_{gn}=\sigma_{g};\;g=1,2,\cdots ,G;\;$$

(9) $$\;\overset{G}{\underset{g=1}{\sum }}\sigma_{g}=1;\;\sigma_{g}=1\;or\;0;\;\gamma_{gn}\;\geq 0;\;n\in g,n\neq o\;if\;g=g^{\prime }.\}\;$$

where φ and γ are the weighting vectors and $\sigma_{g}\left(g=1,2,\cdots ,G\right)$ denotes the subsets’ constraint of the input–output combinations for the *g*-th group. Equations (8) and (9) define the production possible sets of convex and nonconvex metafrontier, respectively, with the following relationship, $P^{c-meta}\subseteq P^{nc-meta}$. Therefore, the efficiency values under the nonconvex metafrontier are not greater than are those under the convex metafrontier. Under the nonconvex metafrontier and super-efficiencies under the assumption of variable returns, the non-oriented and non-radial SBM efficiency values of the *o*-th DMU with reference to the *g*-th group ($o=1,2,\cdots ,N_{g};\;g=1,2,\cdots ,G$) can be obtained by solving the following optimization problem.

$$\;\rho_{g{}^{\prime }o}^{nc-meta*}=\min\frac{1+\frac{1}{M}\;\sum_{m=1}^{M}\frac{s_{mg{}^{\prime }o}^{x}}{x_{mg{}^{\prime }o}}}{1-\frac{1}{R+J}\left(\sum_{r=1}^{R}\frac{s_{rg{}^{\prime }o}^{y}}{y_{rg{}^{\prime }o}}+\sum_{j=1}^{J}\frac{s_{jg{}^{\prime }o}^{b}}{b_{jg{}^{\prime }o}}\right)}\;$$

$$\;s.t.\;\;x_{mg^{\prime }o}-\overset{G}{\underset{g=1}{\sum }}\overset{N_{g}}{\underset{\begin{array}{c} n\in g{}^{\prime },n\neq o\;\\ if\;g=g^{\prime } \end{array}}{\sum }}\gamma_{gn}x_{mgn}+s_{mg^{\prime }o}^{x}\geq 0,\;m=1,2,\cdots ,M;\;$$

$$\;\overset{G}{\underset{g=1}{\sum }}\overset{N_{g}}{\underset{\begin{array}{c} n\in g{}^{\prime },n\neq o\\ if\;g=g{}^{\prime } \end{array}}{\sum }}\gamma_{gn}y_{rgn}-y_{rg^{\prime }o}+s_{rg^{\prime }o}^{y}\geq 0,\;r=1,2,\cdots ,R;\;$$

$$\;b_{jg^{\prime }o}-\overset{G}{\underset{g=1}{\sum }}\overset{N_{g}}{\underset{\begin{array}{c} n\in g{}^{\prime },n\neq o\\ if\;g=g^{\prime } \end{array}}{\sum }}\gamma_{gn}b_{jgn}+s_{jg^{\prime }o}^{b}\geq 0,\;j=1,2,\cdots ,J;\;$$

$$\;1-\frac{1}{R+J}\left(\overset{R}{\underset{r=1}{\sum }}\frac{s_{rg{}^{\prime }o}^{y}}{y_{rg{}^{\prime }o}}+\overset{J}{\underset{j=1}{\sum }}\frac{s_{jg{}^{\prime }o}^{b}}{b_{jg{}^{\prime }o}}\right)\geq \epsilon ;\;$$

$$\;\overset{G}{\underset{g=1}{\sum }}\overset{N_{g}}{\underset{\begin{array}{c} n\in \left(g^{\prime }=1\right),n\neq o\\ if\;g=g^{\prime } \end{array}}{\sum }}\gamma_{gn}=\sigma_{1},\overset{G}{\underset{g=1}{\sum }}\overset{N_{g}}{\underset{\begin{array}{c} n\in \left(g^{\prime }=2\right),n\neq o\\ if\;g=g^{\prime } \end{array}}{\sum }}\gamma_{gn}=\sigma_{2},\cdots ,\overset{G}{\underset{g=1}{\sum }}\overset{N_{g}}{\underset{\begin{array}{c} n\in \left(g^{\prime }=G\right),n\neq o\\ if\;g=g^{\prime } \end{array}}{\sum }}\gamma_{gn}=\sigma_{G};\;$$

(10) $$\;\overset{G}{\underset{g=1}{\sum }}\sigma_{g}=1;\sigma_{g}=1\;or\;0;s^{x},s^{y},s^{b},\gamma \geq 0.\;$$

where ϵ is non-Archimedes infinity, and the constraint $1-\frac{1}{R+J}\left(\sum_{r=1}^{R}\frac{s_{rg{}^{\prime }o}^{y}}{y_{rg{}^{\prime }o}}+\sum_{j=1}^{J}\frac{s_{jg{}^{\prime }o}^{b}}{b_{jg{}^{\prime }o}}\right)\geq \epsilon$ ensures that the denominator of the objective function is greater than zero. $s^{x}$, $s^{y}$, and $s^{b}$ represent the slacks of input variables, desirable outputs, and undesirable outputs, respectively. 

To measure the eco-efficiency more comprehensively and accurately, various input and output variables should be considered as much as possible, which are described as follows. In terms of input variables, we adopt the perpetual inventory method provided previously [40] to estimate capital stock as $K_{i,t}=I_{i,t}+\left(1-\delta_{i,t}\right)K_{i,t-1}$, where $K_{i,t}$, $I_{i,t}$, and $\delta_{i,t}$ denote the capital stock, the annual physical capital investment and the depreciation rate of fixed assets of city i at period t. Labor force is proxied by the total employees in each city. Following the method of a past paper [15], we adopt the area of various administrative division to represent land usage. Finally, primary energy consumption is estimated by the energy intensity statistics multiplied by prefecture gross domestic product (GDP) at the corresponding constant or current prices, for the specific calculation procedure see a previous work [16]. In terms of desirable output, throughout this paper, the real gross domestic product (GDP) is chosen with data values at constant 2010 prices wherever applicable. As for undesirable output, we mainly focus on environmental pollutants, especially the industrial pollutants due to the issue of data availability. We select four types of emissions, i.e., CO_2_ emissions, SO_2_ emissions, wastewater emissions, and soot and dust emissions. The estimation method of CO_2_ emissions is derived from a past work [16]. To avoid the influence of the outliers, we construct an aggregation indicator of environmental pollution index (EPI) using entropy method, higher EPI indicates heavier pollution.

#### 2.2.2. Interested Variables

The interested variables are agglomeration dynamic externalities ($ext_{it}$). The authors of a previous paper [28] argued that agglomeration and development of regional industries may bring about three different types of dynamic externality: Marshall–Arrow–Romer (MAR) externalities [41,42,43], Jacobs externalities [44], and Porter externalities [45]. Until now, studies of agglomeration of economic activities have in fact made use of employment and production data as proxies of agglomeration economies. To accurately characterize the relationship between agglomeration dynamic externalities and urban eco-efficiency, MAR externalities, Jacobs externalities, and Porter externalities are calculated based on the employees of 19 industries (the list can be found in Table A1), the number of industrial enterprises, and the total output value of industrial enterprises. The formulas are as follows

(11) $$\;mar_{it}=\underset{jt}{\max}\left(\frac{h_{ijt}}{h_{jt}}\right)\;$$

(12) $$\;jacobs_{it}=\frac{1}{\sum_{j}\left|h_{ijt}-h_{jt}\right|}\;$$

(13) $$\;porter_{it}=\frac{f_{ijt}/v_{ijt}}{\sum_{i}\sum_{j}f_{ijt}/\sum_{i}\sum_{j}v_{ijt}}\;$$

where $h_{ijt}$ denotes the ratio of the number of employees for city i of industry j at period t (10,000 persons) to the total number of urban residents at period t (10,000 persons), $h_{jt}$ is the number of employees of industry j at period t (10,000 persons) compared with the total number of people in the whole sample at period t (10,000 persons); $f_{ijt}$ and $v_{ijt}$ represent the number of industrial enterprises and total output value of industrial enterprises (million CNY), respectively.

#### 2.2.3. Control Variables

Control variables of eco-efficiency were determined based on the IPAT formula [46], which specifies that environmental quality (I) is impacted by population (P), affluence (A), and technology (T). However, this formula does not permit hypothesis testing. Thus, we select seven primary determinants based on the stochastic model (STIRPAT) proposed previously [47]. 

(1) Environmental regulation (*er*). The implementation of local environmental regulation has a significant impact on eco-efficiency [48]. Differences in urban environmental regulations (e.g., command-and-control environmental and market-based environmental regulations) affect the relocation of domestic and foreign-owned enterprises both inter- and intra-cities in China, potentially resulting in a “Race to the Top” and a “Race to the Bottom”. Due to the limitation of data availability, we chose the removal rate of SO_2_ as a proxy variable to examine the impact of command-and-control environmental regulation on urban eco-efficiency. The removal rate of SO_2_ is the ratio between SO_2_ removed and SO_2_ generated, where the former refers to the volume of removed SO_2_ by waste gas treatment facilities and the latter refers to the volume of generated SO_2_ in waste gas resulting from fuel combustion and production processes for a given period of time. Using the entropy method, we can also generate a composition indicator based on the removal rate of SO_2_, the treatment rate of sewage and comprehensive utilization rate of solid wastes for environmental regulation, and the correlation coefficient between the removal rate of SO_2_ and the composite indicator, which reaches 0.964 with a significant level of 1%. Thus, the removal rate of SO_2_ reflects the command-and-control environmental regulation intensity to some extent. It is expected to have a positive impact on eco-efficiency.

(2) Endowment structure (ln*kl*). Capital and labor are arguably the most basic factors of production and are important endowment resources. Previous studies point out that the technological progress of capital-intensive enterprises may offset their negative impact on environmental efficiency. Furthermore, low-carbon merger and acquisition industries with higher eco-efficiency are mainly distributed in capital-intensive industries, followed by labor-intensive industries. A higher capital–labor ratio means a higher level of capital–labor substitution, implying a higher level of automation and industrial production. Consequently, promotion of eco-efficiency may result from the increase in economic output. In this paper, the capital labor ratio (logarithmic value) is used as the proxy variable of the endowment structure which is expected to have a positive effect on eco-efficiency.

(3) Industrial structure (*s_ind*). The adjustment or optimization of industrial structure means that the proportion of primary industry decreases and the proportion of tertiary industry increases. The optimization of the industrial structure is a dynamic process. “Unclean” industrial agglomeration is mainly concentrated in secondary industry, using nonrenewable resources and environmentally unfriendly materials as inputs for production. An increase of the proportion of secondary industry may cause environmental pollution in a region and further affect the eco-efficiency. Therefore, the proportion of the secondary industry cannot be ignored. We employ the shares of secondary industry in GDP (%) to reflect the industrial structure.

(4) FDI (*fdi*). Foreign capital, represented by FDI, provides both impetus and support to regional development from the outside. It provides not only the capital and technology required for economic construction but also employment. However, FDI may also cause environmental pollution and deteriorate the regional environmental quality [49] especially increase wastewater and sulfur dioxide [50], which harms urban eco-efficiency. Moreover, FDI in general induces negative environmental externalities [51]. To comprehensively examine the impact of FDI on regional sustainable development, this paper uses the relative scale of FDI (the proportion of industrial output value of foreign-invested enterprises above a designated size as a share of GDP) and predicts that this factor will have a negative effect on eco-efficiency. 

(5) Technology capacity (*s_tech*). Investment in research and development helps improve the technology and innovation capability of a region. However, there is a long lag between the input of funds and the completion of technological innovation projects and their applications. To study the impact of technology capacity on eco-efficiency, this paper adopts the shares of expenditure for science and technology to total fiscal expenditure to measure a city’s technology capacity. It is expected to have a significant and positive impact on eco-efficiency. 

(6) Fiscal expenditure structure (*s_fiscal*). Theoretically, government expenditure on environmental protection will pose significant effects on energy conservation and emission reduction that a potential routine to improve eco-efficiency. To study how government fiscal expenditure affects urban eco-efficiency, this paper uses the proportion of local fiscal expenditure to GDP as the proxy variable. Note that fiscal expenditure from local government may exert positive or negative impacts on eco-efficiency. 

(7) Advanced industrial structure (*adv_ind*). In this paper, the ratio of the output value of tertiary industry to the output value of secondary industry is taken as a measure of the degree of industrialization. We assume advanced industrial structure has significant positive association with eco-efficiency.

Finally, the descriptive statistics of variables are summarized in Table 1.

### 2.3. Data

Our sample includes 191 prefecture and above-prefecture level cities in China over the period 2003 to 2013, accounting for more than two-thirds of 286 prefecture and above-prefecture level cities. Our sample contains almost all of the representative cities including the provincial capital cities. Gross domestic product (GDP) of selected sample accounted for approximately 83% and 82% of the national GDP in 2003 and 2013, respectively. Therefore, the study sample is rather representative. The data are extracted from many official resources, such as China Environment Yearbook, China Energy Statistical Yearbook, and China City Statistical Yearbook. Table A2 provides the data sources of relevant variables used in this paper.

## 3. Results and Discussion

### 3.1. Estimation Results of Eco-Efficiency

**Figure 1:**
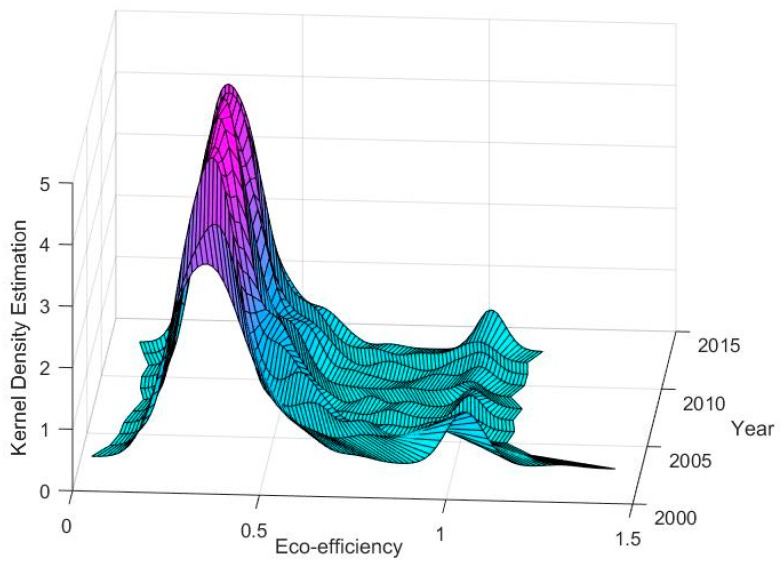
Dynamic evolution of urban eco-efficiency (2003–2013).

We apply the kernel density analysis method to examine the dynamic evolution of China’s urban eco-efficiency from 2003 to 2013. The choice of the kernel density analysis method is based on the following two considerations. On the one hand, it can characterize the overall shape of eco-efficiency. On the other, it enables us to examine the dynamic evolution characteristics of eco-efficiency distribution comprehensively by comparing different periods. As can be seen in Figure 1, the distribution of eco-efficiency has the following characteristics. First, the center point of the eco-efficiency kernel density in 2003 to 2013 is obviously shifted to the left, which means that the eco-efficiency decreased gradually. Second, during our study periods, the peak value of the kernel density function increases continuously, and the main distribution range of eco-efficiency shrinks, which shows that the eco-efficiency distribution in China was increasingly concentrated from 2003 to 2013. Third, longer tailings of kernel density indicate that the differences in eco-efficiency in China are gradually widening. Meanwhile, some cities have higher eco-efficiency (e.g., Beijing and Shanghai) while others are lower eco-efficiency (e.g., Hefei and Changsha). Fourth, an in-depth observation of the peaks of kernel density functions from 2003 to 2013 shows that regional differences in eco-efficiency in China gradually evolved into multilevel differentiation characteristics.

**Figure 2:**
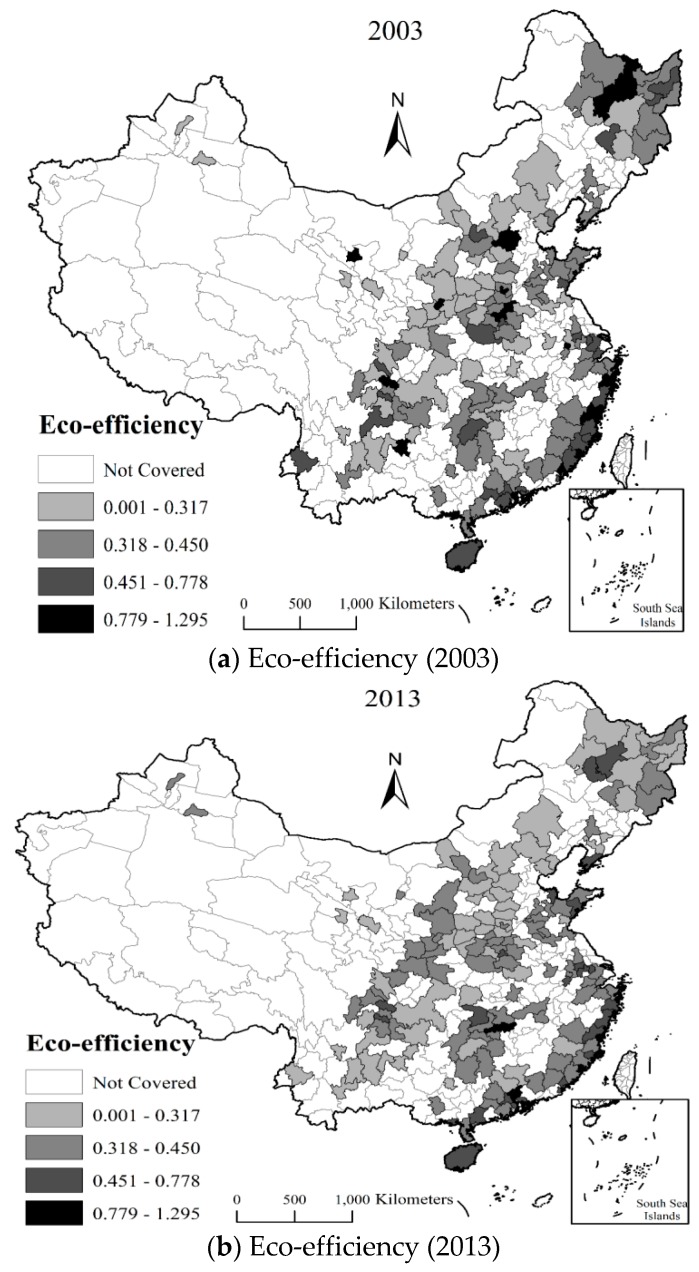
Spatial distribution of urban eco-efficiency in selected years (2003 and 2013).

Next, we further explore the characteristics of urban eco-efficiency that were measured using the proposed model. Both the maximum and the minimum values of eco-efficiency appear in 2003 (Ningde, 1.2950; Yichang, 0.1735). In a certain year, such as 2013, the maximum is reported to be Shenzhen (1.0438), whereas the minimum is reported in Linfen (0.1804). By in-depth analysis of the eco-efficiency growth, compared with 2003, the eco-efficiency of 76 of 191 cities has increased in 2013 and 29 out of 76 cities are located in the eastern region. Figure 2a,b visually depicts the spatial distribution of urban eco-efficiency of two cross-sections in 2003 and 2013, which allows us to observe the spatial evolutions of eco-efficiency. As shown in the graph, cities in the eastern coastal regions maintained high eco-efficiency, while most cities of the central and western regions showed low-value agglomeration. It is clear that the urban eco-efficiency has spatial difference characteristics, which are generally low in the central and western regions while high in the eastern region. Potential explanations for this may be the lagging level of economic development and most heavy industry concentration in the central and western regions, which has not been able to achieve green development for a long time.

### 3.2. Nonlinear Effects of Agglomeration Externalities on Eco-Efficiency

#### 3.2.1. MAR Externalities

**Figure 3:**
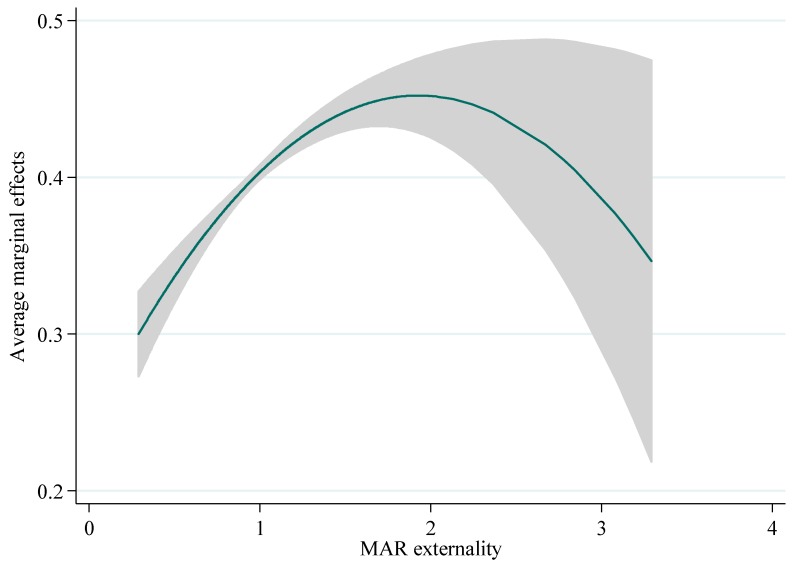
The average marginal effects of MAR externality on eco-efficiency.

Based on model (1) and model (2), we examine the nonlinear effect of MAR externality on urban eco-efficiency. The results are shown in Table 2; control variables are added into the model one by one. As we can see from Table 2, there is a significant inverted U-shaped relationship between MAR externality and eco-efficiency, with a turning point of 1.9293 while all the control variables are considered. When MAR externality crosses 1.9293, the positive effects of MAR externality will decrease; one potential explanation is that congestion effects occur. The average marginal effect of MAR externality on eco-efficiency is 0.1025, which shows that the enhancement of dynamic agglomeration of MAR externality will help to promote urban eco-efficiency in the long run. Nevertheless, over-agglomeration will decrease the promotion of urban eco-efficiency. Figure 3 illustrates the average marginal effect of dynamic MAR externality on urban eco-efficiency, showing that the same inverted U-shaped relationship between agglomeration externality and eco-efficiency also supported. Moreover, all the spatial model estimation results (see Table 3) also show that significant inverted U-shaped relationship between MAR externality and eco-efficiency, and the turning points are ranged from 1.78 to 1.98 with different model specifications.

#### 3.2.2. Jacobs Externalities

**Figure 4:**
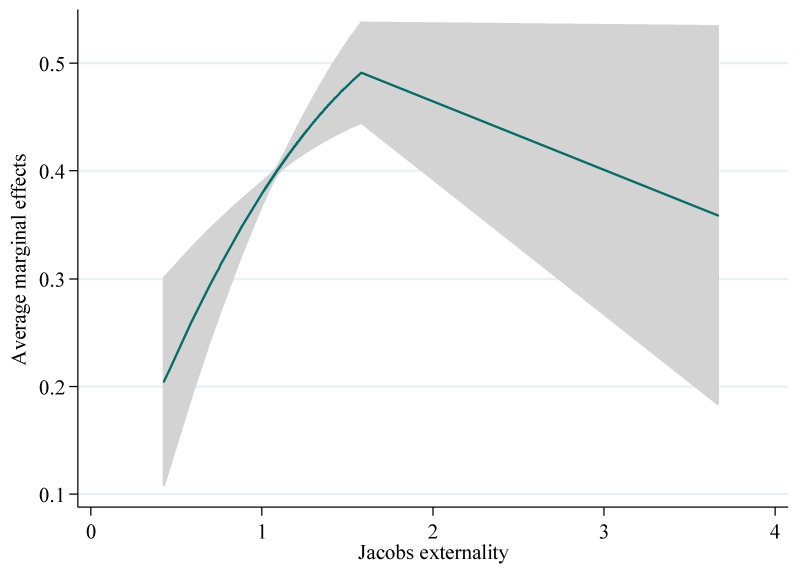
The average marginal effects of Jacobs externality on eco-efficiency.

Based on model (1) and model (2), we continue to investigate the nonlinear effect of Jacobs externality on urban eco-efficiency. The results are shown in Table 4. Estimates of all models show that Jacobs externality also has a significant inverted U-shaped relationship with the urban eco-efficiency, and the turning point is 2.2952 when all control variables are considered at the same time. The average marginal effect of Jacobs externality on eco-efficiency is 0.2317, which indicates that the enhancement of dynamic externality of Jacobs agglomeration also contributes to the improvement of urban eco-efficiency. Similarly, Figure 4 illustrates the average marginal effect of dynamic Jacobs externality on urban eco-efficiency. It also shows an inverted U-shaped form that rises first and falls later. Estimation results of spatial econometrics models (see Table 5) also show that there is a significant inverted U-shaped relationship between the Jacobs externality and urban eco-efficiency, and the turning points are approximately 2.3 even with different model specifications.

#### 3.2.3. Porter Externalities

**Figure 5:**
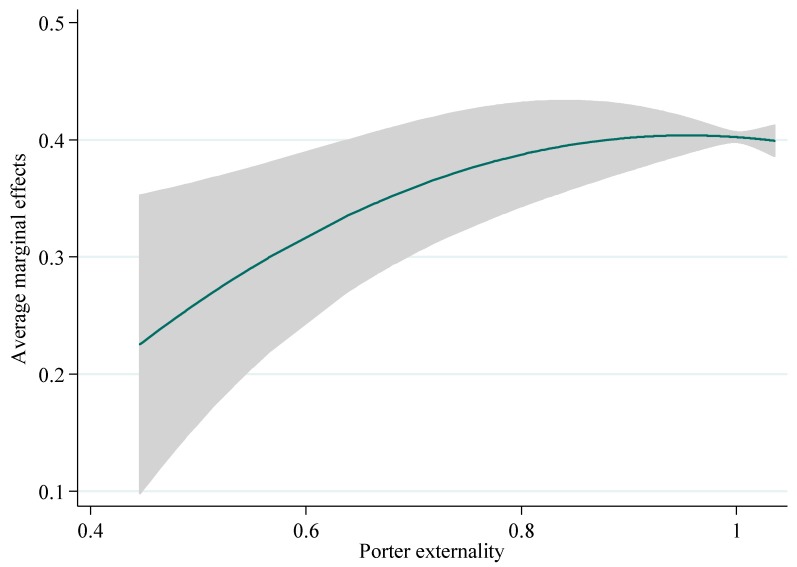
The average marginal effects of the Porter externality.

Based on model (1) and model (2), we continue to explore the nonlinear effects of Porter externality on urban eco-efficiency. The results are listed in Table 6, which show that the Porter externality also exerts significant U-shaped effects on urban eco-efficiency, with a turning point of 0.9539. However, the average marginal effect of the Porter externality on eco-efficiency is −0.0511, which indicates that the weakening of dynamic Porter externality will contribute to the improvement of urban eco-efficiency. Figure 5 depicts the average marginal effect of dynamic Porter externality on urban eco-efficiency, which also has an inverted U-shaped form. Regarding to the spatial model estimation results (see Table 7), the influence of Porter externality on urban eco-efficiency has significant inverted U-shaped relationship, and the turning points range from 0.87 to 0.94.

#### 3.2.4. Robustness Checks

We conduct various robustness checks in this paper. First, we re-estimate the eco-efficiency using the EBM (Epsilon-Based Measure) [52] model that considers nonconvex metafrontier and super-efficiency simultaneously. The regression results show that there is no significant change and the abovementioned conclusions still hold (see Table 8). Second, we consider the dynamic effects and the possible inverse causality relationship between externalities and eco-efficiency, and re-regress the models by adding the lagged term of eco-efficiency, the lagged term of externalities, and the front terms of externalities into the baseline models. As shown in Table 9, the inverted U-shaped relationship between externalities and eco-efficiency is also supported; the conclusions mentioned before are robust. Finally, we employ the algorithm proposed by the authors of a previous paper [53] and confirm that the abovementioned conclusions are robust, estimation results are shown in Table 10. Hence, empirical findings show the effects of agglomerative dynamic externalities on urban eco-efficiency have a significant inverted U-shape. All of the robustness checks indicate that the dynamic external externalities strongly and positively impact urban eco-efficiency until they reach some thresholds. After that, the impact begins to decrease.

## 4. Conclusions 

Eco-efficiency improvement essentially requires the synergistic development of resources, the environment, and the economy. Due to its comprehensive nature of eco-efficiency, numerous factors may impact eco-efficiency and it is impossible to include all of them. Thus, it is important to identify the key influence factors of eco-efficiency. To this end, in this paper, we study the impacts of agglomeration externalities on eco-efficiency. Overall, we find that appropriate agglomeration is beneficial to promote eco-efficiency. However, excessive agglomeration may negatively impact eco-efficiency because of the congestion effects. Specifically, in this paper, we extend the convex metafrontier methodology extended to a nonconvex one, and measure the eco-efficiency of 191 prefecture-level cities in China. We further investigate the nonlinear effects of agglomeration externalities on eco-efficiency using both nonspatial and spatial econometric methods. Furthermore, we also conduct various tests and show the robustness of our results. Our main findings are summarized as follows.

First, our results from the kernel density estimation show that urban eco-efficiency gradually decreased from 2003 to 2013. Furthermore, the distribution of urban eco-efficiency in China became increasingly concentrated from 2003 to 2013. In addition, when observing the peaks of the kernel density estimations, we found that the regional differences of urban eco-efficiency gradually evolve into multilevel differentiation characteristics from the earlier two-level differentiation. One potential explanation is that the increasing level of agglomeration has led to the emergence of several eco-efficiency growth poles, along with multilevel differentiation characteristics of eco-efficiency. Therefore, a policy implication is that narrowing the gap in eco-efficiency between cities with lower eco-efficiency and those with higher eco-efficiency will be conducive to the overall improvement of urban eco-efficiency.

Second, the temporal and spatial evolution of urban eco-efficiency shows that eco-efficiency has a spatial distribution pattern. The eco-efficiency of cities in China’s eastern regions is the highest, followed by the central-western regions where the eco-efficiency may possess the path-dependence and locked-in characteristics. Therefore, it is necessary to further pursue sustainable development and improve the eco-efficiency of cities in China’s central-western regions.

Third, both MAR externality, Jacobs externality, and Porter externality lead to a significant inverted U-shaped relationship with urban eco-efficiency; this empirical finding is robust to different measures of efficiency and model specifications. Empirical results also show that the endowment structure has a negative effect on urban eco-efficiency. On the other hand, the strengthening of environmental regulation, technology capacity, and industrialization contribute to the improvement of urban eco-efficiency. Consequently, the agglomeration externalities exert significant nonlinear effects on eco-efficiency. Specifically, agglomeration externalities have a significant and positive relationship with eco-efficiency before an agglomeration threshold. After that, their relationship becomes significant but negative. In terms of policy implication, it indicates that policy-makers need to control the degree of agglomeration to avoid the congestion effect due to excessive industrial agglomeration. To better promote urban eco-efficiency, policy-makers need to optimize the endowment structure, for example, by the efficient use and rational allocation of natural resources. For instance, Yichun city, located in Heilongjiang province and listed as a resource-based city, is not sustainable if it relies too greatly on the industrial structure of forestry natural resources. Moreover, if the exploitation of forestry resources is exhausted, it is not conducive to the sustainable development of the social economy. Given this, efforts should be made to strengthen environmental regulation, increase investment on science and technology, and facilitate industrial restructuring to effectively and efficiently promote urban eco-efficiency. 

There are several future research directions. First, the time span can be increased to cover a longer period of time, and more information and data can be used to further analyze urban eco-efficiency, such as convergence analysis from both nonspatial and spatial perspectives. Second, the proposed DEA model in this paper can be extended to measure and compare productivity changes for cities in different countries under the framework of the Malmquist–Luenberger productivity indicator. With the same metafrontier, these indicators are comparable and can provide insightful information. Third, it is important to further investigate the contribution of technological development to the eco-efficiency. With Malmquist–Luenberger productivity indicator defined, we can decompose it into efficiency change (EC), technological change (TC), and technological ratio gap change (TGRC) and calculate the shares of EC, TC, and TGRC in eco-efficiency growth, along with identifying the main sources of eco-efficiency growth.

## Figures and Tables

**Table 1:** Descriptive statistics.

Variables	Observations	Mean	Standard Deviation	Minimum	Maximum
*ee*	2101	0.40344	0.1734	0.1735	1.2950
*mar*	2101	1.0239	0.3360	0.2891	3.2944
*jacobs*	2101	1.0907	0.1080	0.4273	3.6675
*porter*	2101	0.9909	0.0703	0.4460	1.0349
*er*	2101	0.3799	0.2634	0.0100	0.9900
ln*kl*	2101	3.7291	0.6718	1.5839	5.4519
*s_ind*	2101	0.4965	0.1160	0.1570	0.9097
*fdi*	2101	0.1672	0.1770	0.0000	0.8554
*s_tech*	2101	9.2000	1.8684	−2.0402	14.7620
*s_fiscal*	2101	0.1354	0.0774	0.0154	1.5642
*adv_ind*	2101	0.8191	0.4199	0.0943	3.4431

**Table 2:** Estimation results of MAR externality and urban eco-efficiency.

Variables	(1)	(2)	(3)	(4)	(5)	(6)	(7)
*mar*	0.1435 ***	0.2342 ***	0.2180 ***	0.2115 ***	0.2151 ***	0.2153 ***	0.2184 ***
	(3.7178)	(6.1650)	(5.7168)	(5.5268)	(5.6864)	(5.7151)	(5.8553)
*mar × mar*	−0.0501 ***	−0.0618 ***	−0.0576 ***	−0.0552 ***	−0.0549 ***	−0.0550 ***	−0.0566 ***
	(−3.2915)	(−4.2044)	(−3.9205)	(−3.7430)	(−3.7606)	(−3.7858)	(−3.9368)
*er*	0.0245 **	0.0356 ***	0.0315 ***	0.0315 ***	0.0323 ***	0.0277 **	0.0272 **
	(2.0914)	(3.1396)	(2.7687)	(2.7768)	(2.8746)	(2.4631)	(2.4444)
*lnkl*		−0.1204 ***	−0.1073 ***	−0.1054 ***	−0.1107 ***	−0.1086 ***	−0.1094 ***
		(−11.9921)	(−10.0859)	(−9.8688)	(−10.4524)	(−10.2909)	(−10.4739)
*s_ind*			−0.0020 ***	−0.0020 ***	−0.0014 **	−0.0012 **	0.0033 ***
			(−3.6538)	(−3.6597)	(−2.5519)	(−2.2403)	(3.6852)
*fdi*				−0.0650 *	−0.0599 *	−0.0564	−0.0535
				(−1.8433)	(−1.7180)	(−1.6230)	(−1.5547)
*s_tech*					0.0260 ***	0.0248 ***	0.0228 ***
					(6.6858)	(6.3891)	(5.9027)
*s_fiscal*						−0.1875 ***	−0.1723 ***
						(−4.1328)	(−3.8310)
*adv_ind*							0.1305 ***
							(6.2904)
Constant	0.3524 ***	0.6168 ***	0.6845 ***	0.6939 ***	0.4970 ***	0.5105 ***	0.1980 ***
	(14.3915)	(19.0922)	(18.4234)	(18.5142)	(10.4993)	(10.8041)	(2.9028)
Observations	2101	2101	2101	2101	2101	2101	2101
R-squared	0.0570	0.1235	0.1296	0.1312	0.1512	0.1588	0.1761

Notes: (1) t-statistics in parentheses; (2) *** *p* < 0.01, ** *p* < 0.05, * *p* < 0.1.

**Table 3:** Spatial effects of MAR externality on eco-efficiency.

Variables	(1)	(2)	(3)	(4)
	SAR	SEM	SDM	SAC
*mar*	0.1357 ***	0.2096 ***	0.2195 ***	0.2129 ***
	(3.8948)	(5.8970)	(6.1386)	(6.0067)
*mar* × *mar*	−0.0381 ***	−0.0533 ***	−0.0554 ***	−0.0544 ***
	(−2.7587)	(−3.9334)	(−4.0550)	(−4.0190)
*er*	0.0412 ***	0.0305 ***	0.0275 **	0.0298 ***
	(3.9259)	(2.8544)	(2.5539)	(2.7971)
ln*kl*	−0.0341 ***	−0.1024 ***	−0.1178 ***	−0.1062 ***
	(−6.1976)	(−9.4003)	(−10.4331)	(−9.8139)
*s_ind*	0.0031 ***	0.0033 ***	0.0038 ***	0.0033 ***
	(3.5797)	(3.6462)	(3.9384)	(3.6293)
*fdi*	−0.0939 ***	−0.0759 **	−0.0768 **	−0.0758 **
	(−2.8561)	(−2.3043)	(−2.3158)	(−2.3055)
*s_tech*	0.0072 ***	0.0189 ***	0.0194 ***	0.0193 ***
	(5.0399)	(5.6194)	(5.1221)	(5.6206)
*s_fiscal*	−0.0981 **	−0.1442 ***	−0.1522 ***	−0.1498 ***
	(−2.3284)	(−3.3580)	(−3.5051)	(−3.4871)
*adv_ind*	0.1520 ***	0.1374 ***	0.1408 ***	0.1367 ***
	(7.6580)	(6.8041)	(6.8227)	(6.7770)
*W* × *mar*			−0.3485	
			(−1.2775)	
*W* × *mar* × *mar*			0.1024	
			(0.9318)	
*W* × *er*			0.0269	
			(0.4266)	
*W* × ln*kl*			0.1132 ***	
			(4.8679)	
*W* × *s_ind*			−0.0107 *	
			(−1.9159)	
*W* × *fdi*			0.1147	
			(0.5592)	
*W* × *s_tech*			−0.0187 ***	
			(−4.4343)	
*W* × *s_fiscal*			0.0296	
			(0.1263)	
*W* × *adv_ind*			−0.3241 **	
			(−2.1923)	
*ρ*	0.5872 ***		0.6628 ***	−0.2671 *
	(8.4240)		(9.2129)	(−1.7694)
*λ*		0.8276 ***		0.8464 ***
		(21.8569)		(24.2155)
Observations	2101	2101	2101	2101
R-squared	0.0003	0.0023	0.0056	0.0023

Notes: (1) z-statistics in parentheses; (2) *** *p* < 0.01, ** *p* < 0.05, * *p* < 0.1.

**Table 4:** Estimation results of Jacobs externality and urban eco-efficiency.

Variables	(1)	(2)	(3)	(4)	(5)	(6)	(7)
*jacobs*	0.7372 ***	0.6376 ***	0.6036 ***	0.5811 ***	0.5545 ***	0.5357 ***	0.4416 ***
	(6.3861)	(5.6131)	(5.3276)	(5.1026)	(4.9114)	(4.7592)	(3.9080)
*jacobs* × *jacobs*	−0.1587 ***	−0.1413 ***	−0.1346 ***	−0.1277 ***	−0.1201 ***	−0.1158 ***	−0.0962 ***
	(−5.7847)	(−5.2476)	(−5.0143)	(−4.7129)	(−4.4732)	(−4.3240)	(−3.5882)
*er*	0.0282 **	0.0377 ***	0.0325 ***	0.0325 ***	0.0330 ***	0.0286 **	0.0282 **
	(2.4270)	(3.2996)	(2.8476)	(2.8428)	(2.9172)	(2.5258)	(2.5109)
ln*kl*		−0.0836 ***	−0.0709 ***	−0.0693 ***	−0.0726 ***	−0.0707 ***	−0.0724 ***
		(−9.0490)	(−7.3534)	(−7.1640)	(−7.5643)	(−7.3945)	(−7.6238)
*s_ind*			−0.0024 ***	−0.0024 ***	−0.0019 ***	−0.0017 ***	0.0024 **
			(−4.4114)	(−4.4039)	(−3.4254)	(−3.1365)	(2.5776)
*fdi*				−0.0660 *	−0.0634 *	−0.0607 *	−0.0601 *
				(−1.8405)	(−1.7835)	(−1.7138)	(−1.7106)
*s_tech*					0.0240 ***	0.0229 ***	0.0212 ***
					(6.1254)	(5.8496)	(5.4302)
*s_fiscal*						−0.1788 ***	−0.1667 ***
						(−3.9037)	(−3.6642)
*adv_ind*							0.1174 ***
							(5.5255)
Constant	−0.1712 *	0.1560	0.2610 **	0.2835 ***	0.1228	0.1514	−0.0454
	(−1.8071)	(1.5664)	(2.5607)	(2.7632)	(1.1700)	(1.4446)	(−0.4133)
Observations	2101	2101	2101	2101	2101	2101	2101
R-squared	0.0708	0.1092	0.1183	0.1199	0.1370	0.1439	0.1575

Notes: (1) t-statistics in parentheses; (2) *** *p* < 0.01, ** *p* < 0.05, * *p* < 0.1.

**Table 5:** Spatial effects of Jacobs externality on eco-efficiency.

Variables	(1)	(2)	(3)	(4)
	SAR	SEM	SDM	SAC
*jacobs*	0.4645 ***	0.4434 ***	0.4237 ***	0.4407 ***
	(4.3217)	(4.1583)	(3.9687)	(4.1377)
*jacobs* × *jacobs*	−0.0999 ***	−0.0959 ***	−0.0915 ***	−0.0953 ***
	(−3.9169)	(−3.7890)	(−3.6112)	(−3.7720)
*er*	0.0381 ***	0.0321 ***	0.0301 ***	0.0316 ***
	(3.6414)	(2.9873)	(2.7705)	(2.9406)
ln*kl*	−0.0290 ***	−0.0668 ***	−0.0802 ***	−0.0700 ***
	(−5.3511)	(−7.3375)	(−7.6727)	(−7.3879)
*s_ind*	0.0023 ***	0.0023 **	0.0026 ***	0.0023 **
	(2.6026)	(2.4782)	(2.6257)	(2.4656)
*fdi*	−0.0872 ***	−0.0790 **	−0.0830 **	−0.0790 **
	(−2.6220)	(−2.3430)	(−2.4414)	(−2.3443)
*s_tech*	0.0065 ***	0.0164 ***	0.0190 ***	0.0170 ***
	(4.5769)	(5.1024)	(4.9530)	(5.1019)
*s_fiscal*	−0.1096 ***	−0.1470 ***	−0.1564***	−0.1513 ***
	(−2.6043)	(−3.4023)	(−3.5625)	(−3.4895)
*adv_ind*	0.1310 ***	0.1251 ***	0.1292 ***	0.1246 ***
	(6.5071)	(6.0552)	(6.0911)	(6.0292)
*W* × *jacobs*			−1.2180	
			(−1.3419)	
*W* × *jacobs* × *jacobs*			0.2487	
			(1.1219)	
*W* × *er*			0.0295	
			(0.4672)	
*W* × ln*kl*			0.0726 ***	
			(3.0637)	
*W* × *s_ind*			−0.0087 *	
			(−1.6580)	
*W* × *fdi*			0.1049	
			(0.4906)	
*W* × *s_tech*			−0.0190 ***	
			(−4.4550)	
*W* × *s_fiscal*			0.0742	
			(0.3144)	
*W* × *adv_ind*			−0.3250 **	
			(−2.2213)	
*ρ*	0.6135 ***		0.6479 ***	−0.1765
	(8.9971)		(8.8285)	(−1.1106)
*λ*		0.7570 ***		0.7882 ***
		(14.5573)		(15.1576)
Observations	2101	2101	2101	2101
R-squared	0.0000	0.0008	0.0004	0.0008

Notes: (1) z-statistics in parentheses; (2) *** *p* < 0.01, ** *p* < 0.05, * *p* < 0.1.

**Table 6:** Estimation results of the Porter externality and urban eco-efficiency.

Varibles	(1)	(2)	(3)	(4)	(5)	(6)	(7)
*porter*	2.2885 ***	2.0824 ***	2.0929 ***	2.0566 ***	1.7181 ***	1.3774 **	1.3198 **
	(3.3876)	(3.1569)	(3.1913)	(3.1383)	(2.6359)	(2.1008)	(2.0317)
*porter* × *porter*	−1.3865 ***	−1.1325 ***	−1.1335 ***	−1.1136 ***	−0.9131 **	−0.7048 *	−0.6918 *
	(−3.3932)	(−2.8337)	(−2.8531)	(−2.8049)	(−2.3129)	(−1.7745)	(−1.7580)
*er*	0.0244 **	0.0347 ***	0.0290 **	0.0291 **	0.0302 ***	0.0264 **	0.0261 **
	(2.0766)	(3.0156)	(2.5218)	(2.5273)	(2.6481)	(2.3164)	(2.3109)
ln*kl*		−0.0925 ***	−0.0783 ***	−0.0764 ***	−0.0797 ***	−0.0782 ***	−0.0785 ***
		(−9.7760)	(−7.9459)	(−7.7331)	(−8.1300)	(−7.9924)	(−8.0961)
*s_ind*			−0.0026 ***	−0.0026 ***	−0.0021 ***	−0.0019 ***	0.0025 ***
			(−4.8346)	(−4.8205)	(−3.8407)	(−3.5369)	(2.7683)
*fdi*				−0.0780 **	−0.0740 **	−0.0711 **	−0.0688 **
				(−2.1993)	(−2.1068)	(−2.0305)	(−1.9827)
*s_tech*					0.0237 ***	0.0227 ***	0.0208 ***
					(6.0007)	(5.7711)	(5.3108)
*s_fiscal*						−0.1765 ***	−0.1619 ***
						(−3.8061)	(−3.5181)
*adv_ind*							0.1277 ***
							(6.0631)
Constant	−0.4550 *	−0.2377	−0.1643	−0.1409	−0.1820	−0.0379	−0.3003
	(−1.6504)	(−0.8804)	(−0.6110)	(−0.5242)	(−0.6833)	(−0.1415)	(−1.1157)
Observations	2101	2101	2101	2101	2101	2101	2101
R-squared	0.0555	0.1009	0.1118	0.1141	0.1306	0.1372	0.1537

Notes: (1) t-statistics in parentheses; (2) *** *p* < 0.01, ** *p* < 0.05, * *p* < 0.1.

**Table 7:** Spatial effects of the Porter externality on eco-efficiency.

Variables	(1)	(2)	(3)	(4)
	SAR	SEM	SDM	SAC
*porter*	1.5022 **	1.3467 **	1.4023 **	1.3593 **
	(2.4272)	(2.1408)	(2.1950)	(2.1626)
*porter* × *porter*	−0.8672 **	−0.7198 *	−0.7473 *	−0.7218 *
	(−2.3192)	(−1.8914)	(−1.9359)	(−1.8986)
*er*	0.0363 ***	0.0312 ***	0.0290 ***	0.0306 ***
	(3.4493)	(2.8901)	(2.6445)	(2.8376)
ln*kl*	−0.0308 ***	−0.0714 ***	−0.0860 ***	−0.0754 ***
	(−5.6670)	(−7.5397)	(−8.0963)	(−7.6883)
*s_ind*	0.0026 ***	0.0026 ***	0.0030 ***	0.0025 ***
	(2.9425)	(2.7724)	(3.0362)	(2.7481)
*fdi*	−0.0962 ***	−0.0887 ***	−0.0924 ***	−0.0887 ***
	(−2.9199)	(−2.6658)	(−2.7567)	(−2.6678)
*s_tech*	0.0065 ***	0.0164 ***	0.0187 ***	0.0171 ***
	(4.5671)	(5.0966)	(4.8585)	(5.1068)
*s_fiscal*	−0.0999 **	−0.1412 ***	−0.1535 ***	−0.1464 ***
	(−2.3472)	(−3.2300)	(−3.4596)	(−3.3397)
*adv_ind*	0.1445 ***	0.1373 ***	0.1434 ***	0.1365 ***
	(7.2570)	(6.7067)	(6.8422)	(6.6639)
*W* × *porter*			−1.8517	
			(−0.3943)	
*W* × *porter* × *porter*			1.2462	
			(0.4217)	
*W* × *er*			0.0021	
			(0.0333)	
*W* × ln*kl*			0.0893 ***	
			(3.7885)	
*W* × *s_ind*			−0.0106 *	
			(−1.8671)	
*W* × *fdi*			0.1215	
			(0.5800)	
*W* × *s_tech*			−0.0185 ***	
			(−4.3363)	
*W* × *s_fiscal*			0.1069	
			(0.4165)	
*W* × *adv_ind*			−0.3703 **	
			(−2.3935)	
*ρ*	0.5971 ***		0.6470 ***	−0.2124
	(8.5988)		(8.8090)	(−1.3408)
*λ*		0.7614 ***		0.7960 ***
		(14.7100)		(16.0125)
Observations	2101	2101	2101	2101
R-squared	0.0007	0.0033	0.0071	0.0034

Notes: (1) z-statistics in parentheses; (2) *** *p* < 0.01, ** *p* < 0.05, * *p* < 0.1.

**Table 8:** Robustness check: eco-efficiency alternative measure.

Vaaribles	(1)	(2)	(3)
*mar*	0.1900 ***		
	(5.8564)		
*mar* × *mar*	−0.0383 ***		
	(−3.0635)		
*jacobs*		0.2147 **	
		(2.1597)	
*jacobs* × *jacobs*		−0.0471 **	
		(−1.9975)	
*porter*			1.1929 **
			(2.1012)
*porter* × *porter*			−0.5063
			(−1.4722)
*er*	0.0302 ***	0.0299 ***	0.0281 ***
	(3.1159)	(3.0228)	(2.8466)
ln*kl*	−0.1552 ***	−0.1148 ***	−0.1233 ***
	(−17.0801)	(−13.7358)	(−14.5606)
*s_ind*	0.0057 ***	0.0048 ***	0.0047 ***
	(7.2134)	(6.0233)	(5.9408)
*fdi*	−0.0461	−0.0553 *	−0.0579 *
	(−1.5399)	(−1.7897)	(−1.9083)
*s_tech*	0.0264 ***	0.0246 ***	0.0241 ***
	(7.8681)	(7.1695)	(7.0526)
*s_fiscal*	−0.2349 ***	−0.2327 ***	−0.2279 ***
	(−6.0049)	(−5.8108)	(−5.6672)
*adv_ind*	0.1257 ***	0.1203 ***	0.1213 ***
	(6.9670)	(6.4354)	(6.5868)
Constant	0.4126 ***	0.3288 ***	−0.1452
	(6.9519)	(3.3992)	(−0.6173)
Observations	2101	2101	2101
R-squared	0.2723	0.2384	0.2454

Notes: (1) t-statistics in parentheses; (2) *** *p* < 0.01, ** *p* < 0.05, * *p* < 0.1.

**Table 9:** Robustness check: dynamic effects and inverse causality tests.

Variables	(1)	(2)	(3)	(4)	(5)	(6)	(7)	(8)	(9)
	Adding Lag Term of Eco-Efficiency			Adding Lag Terms of Externalities			Adding Front Terms of Externalities		
Lagged (*EE*)	0.6836 ***	0.6837 ***	0.6860 ***						
	(51.0342)	(52.0274)	(52.1612)						
*mar*	0.0148			0.2358 ***			0.1785 ***		
	(0.6914)			(6.4432)			(4.8017)		
*mar* × *mar*	−0.0027			−0.0752 ***			−0.0515 ***		
	(−0.3356)			(−5.1120)			(−3.5162)		
*jacobs*		0.1823 ***			1.1729 ***			0.3367 ***	
		(2.9917)			(3.5881)			(3.0344)	
*jacobs* × *jacobs*		−0.0405 ***			−0.4532 ***			−0.0728 ***	
		(−2.8052)			(−3.0639)			(−2.7876)	
*porter*			0.1820			0.6265			1.1749 *
			(0.4688)			(1.0891)			(1.6601)
*porter* × *porter*			−0.0628			−0.3690			−0.7221 *
			(−0.2653)			(−1.0585)			(−1.6757)
*er*	0.0213 ***	0.0213 ***	0.0209 ***	0.0187 *	0.0239 **	0.0232 **	0.0187	0.0177	0.0162
	(3.3853)	(3.3895)	(3.3134)	(1.8659)	(2.3592)	(2.2757)	(1.5667)	(1.4741)	(1.3429)
ln*kl*	−0.0278 ***	−0.0233 ***	−0.0261 ***	−0.0917 ***	−0.0749 ***	−0.0735 ***	−0.0739 ***	−0.0609 ***	−0.0616 ***
	(−4.2614)	(−4.0918)	(−4.4399)	(−9.8755)	(−8.2368)	(−8.0815)	(−6.8337)	(−5.7010)	(−5.7375)
*s_ind*	0.0024 ***	0.0022 ***	0.0023 ***	0.0036 ***	0.0029 ***	0.0032 ***	0.0018 *	0.0012	0.0015
	(4.5221)	(4.1705)	(4.3404)	(4.2593)	(3.4189)	(3.6392)	(1.8950)	(1.2755)	(1.5137)
*fdi*	−0.0674 ***	−0.0630 ***	−0.0678 ***	−0.0899 ***	−0.0954 ***	−0.0987 ***	0.0654	0.0513	0.0441
	(−3.4860)	(−3.2300)	(−3.5238)	(−2.9328)	(−3.0850)	(−3.1793)	(1.2119)	(0.9448)	(0.8108)
*s_tech*	0.0031	0.0030	0.0029	0.0174 ***	0.0171 ***	0.0164 ***	0.0177 ***	0.0172 ***	0.0168 ***
	(1.4083)	(1.3435)	(1.3169)	(4.9700)	(4.8251)	(4.6155)	(4.5768)	(4.4189)	(4.2912)
*s_fiscal*	0.0345	0.0364	0.0352	−0.0931 **	−0.0908 **	−0.0892 **	−0.6770 ***	−0.6677 ***	−0.6568 ***
	(1.3920)	(1.4730)	(1.4089)	(−2.3757)	(−2.2922)	(−2.2276)	(−9.1757)	(−8.9903)	(−8.7431)
*adv_ind*	0.0598 ***	0.0545 ***	0.0582 ***	0.1336 ***	0.1190 ***	0.1313 ***	0.0973 ***	0.0831 ***	0.0948 ***
	(4.8843)	(4.4191)	(4.7449)	(6.9050)	(5.9770)	(6.5399)	(4.4150)	(3.6867)	(4.2614)
Constant	−0.0077	−0.1446 **	−0.1115	0.0904	−0.4939 **	−0.0314	0.2963 ***	0.1509	−0.0365
	(−0.1731)	(−2.2645)	(−0.6986)	(1.2686)	(−2.5725)	(−0.1320)	(4.0137)	(1.3640)	(−0.1252)
Observations	1910	1910	1910	1910	1910	1910	1910	1910	1910
R-squared	0.6681	0.6696	0.6682	0.1601	0.1434	0.1350	0.1879	0.1760	0.1728

Notes: (1) t-statistics in parentheses; (2) *** *p* < 0.01, ** *p* < 0.05, * *p* < 0.1.

**Table 10:** Robustness check: alternative approach by Simar and Wilson (2007).

Variables	(1)	(2)	(3)
*mar*	0.0575 ***		
	(4.6039)		
*mar* × *mar*	−0.1027 ***		
	(−3.4084)		
*jacobs*		0.0771 ***	
		(3.2387)	
*jacobs* × *jacobs*		−0.4410 ***	
		(−6.0581)	
*porter*			0.4212
			(1.1068)
*porter* × *porter*			−0.2649
			(−1.1299)
*er*	−0.0162	−0.0201 *	−0.0198 *
	(−1.4483)	(−1.7699)	(−1.7694)
ln*kl*	−0.0007	0.0030	0.0043
	(−0.1266)	(0.5833)	(0.8308)
*s_ind*	0.0010 **	0.0008 *	0.0010 **
	(2.2315)	(1.9517)	(2.2093)
*fdi*	0.2608 ***	0.2901 ***	0.2748 ***
	(15.2070)	(17.2982)	(16.2549)
*s_tech*	−0.0009	0.0023	−0.0016
	(−0.5454)	(1.3780)	(−0.9603)
*s_fiscal*	−0.0906 **	−0.1621 ***	−0.0931 **
	(−2.1876)	(−3.9534)	(−2.2885)
*adv_ind*	0.0371 ***	0.0425 ***	0.0395 ***
	(3.0956)	(3.6846)	(3.2518)
Constant	0.3216 ***	0.6384 ***	0.1114
	(8.8120)	(10.5686)	(0.7184)
Observations	2101	2101	2101

Notes: (1) z-statistics in parentheses; (2) *** *p* < 0.01, ** *p* < 0.05, * *p* < 0.1; and (3) the z-statistics and *p*-value have generated from bootstrap method using 2000 replications.

## Appendix A

**Table A1:** List of 19 industries in China.

No.	Industries
1	Primary Industry
2	Mining
3	Manufacturing
4	Production and Distribution of Electricity, Gas and Water
5	Construction
6	Wholesale and Retail Trades
7	Traffic, Transport, Storage and Post
8	Hotels and Catering Services
9	Information Transmission, Computer Services and Software
10	Financial Intermediation
11	Real Estate
12	Leasing and Business Services
13	Scientific Research, Technical Service and Geologic Prospecting
14	Management of Water Conservancy, Environment
15	Services to Households and Other Services
16	Education
17	Health, Social Security and Social Welfare
18	Culture, Sports and Entertainment
19	Public Management and Social Organization

Notes: (1) Secondary industry included NO. 2–NO. 5 and tertiary industry included NO. 6–NO. 19. (2) Data sources: China City Statistical Yearbook, 2017.

## Appendix B

**Table A2:** Data sources.

	Variable	Sources
Panel A: DEA model		
Input	Capital	China City Statistical Yearbook and China Statistical Yearbook
Input	Labor	China City Statistical Yearbook
Input	Land	China City Statistical Yearbook
Input	Energy	GDP energy intensity (manually collected from various official documents) multiplied by GDP, China Energy Statistical Yearbook
Desirable output	GDP	China City Statistical Yearbook
Undesirable output	EPI	China City Statistical Yearbook and China Environment Yearbook
Panel B: Econometric model		
Dependent variable	*ee*	Measured by Model (10)
Interest variables	*mar*	Measured by Model (11), original data from China City Statistical Yearbook
	*jacobs*	Measured by Model (12), original data from China City Statistical Yearbook
	*porter*	Measured by Model (13), original data from China City Statistical Yearbook
Control variables	*er*	China City Statistical Yearbook and China Environment Yearbook
	ln*kl*	China City Statistical Yearbook
	*s_ind*	China City Statistical Yearbook
	*fdi*	China City Statistical Yearbook and China Statistical Yearbook
	*s_tech*	China City Statistical Yearbook
	*s_fiscal*	China City Statistical Yearbook
	*adv_ind*	China City Statistical Yearbook

## References

[1] Verfaillie H.A., Bidwell R. (2000). Measuring Eco-Efficiency: A Guide to Reporting Company Performance.

[2] OECD Eco-Efficiency. Proceedings of the Conference on Resource Efficiency.

[3] Waste from Electrical and Electronic Equipment (WEEE). https://www.eea.europa.eu/data-and-maps/indicators/waste-electrical-and-electronic-equipment/assessment-1.

[4] Beltrán-Esteve M., Gómez-Limón J.A., Picazo-Tadeo A.J., Reig-Martínez E. (2014). A metafrontier directional distance function approach to assessing eco-efficiency. J. Prod. Anal..

[5] Orea L., Wall A. (2017). A parametric approach to estimating eco-efficiency. J. Agric. Eco..

[6] Deng X., Gibson J. (2018). Sustainable land use management for improving land eco-efficiency: A case study of Hebei, China. Ann. Oper. Res..

[7] Kuosmanen T., Kortelainen M. (2005). Measuring eco-efficiency of production with data envelopment analysis. J. Ind. Ecol..

[8] Zhang B., Bi J., Fan Z., Ge J. (2008). Eco-efficiency analysis of industrial system in China: A data envelopment analysis approach. Ecol. Eco..

[9] Chen C.M. (2014). Evaluating eco-efficiency with data envelopment analysis: An analytical reexamination. Ann. Oper. Res..

[10] Rashidi K., Saen R.F. (2015). Measuring eco-efficiency based on green indicators and potentials in energy saving and undesirable output abatement. Energy Eco..

[11] Arabi B., Munisamy S., Emrouznejad A., Toloo M., Ghazizadeh M.S. (2016). Eco-efficiency considering the issue of heterogeneity among power plants. Energy.

[12] Beltrán-Esteve M., Reig-Martínez E., Estruch-Guitart V. (2017). Assessing eco-efficiency: A metafrontier directional distance function approach using life cycle analysis. Environ. Impact Assess. Rev..

[13] Fan Y., Bai B., Qiao Q., Kang P., Zhang Y. (2017). Study on eco-efficiency of industrial parks in China based on data envelopment analysis. J. Environ. Manag..

[14] Yue S., Yang Y., Pu Z. (2017). Total-factor ecology efficiency of regions in China. Ecol. Indic..

[15] Huang J., Xia J., Yu Y., Zhang N. (2018). Composite eco-efficiency indicators for China based on data envelopment analysis. Ecol. Indic..

[16] Huang J., Yu Y., Ma C. (2018). Energy Efficiency Convergence in China: Catch-Up, Lock-In and Regulatory Uniformity. Environ. Res. Eco..

[17] Moutinho V., Madaleno M., Robaina M., Villar J. (2018). Advanced scoring method of eco-efficiency in European cities. Environ. Sci. Pollut. Res. Int..

[18] Fernández C., Koop G., Steel M.F.J. (2002). Multiple-output production with undesirable outputs. Publ. Am. Stat. Assoc..

[19] Battese G.E., Rao D.P., O’Donnell C.J. (2004). A metafrontier production function for estimation of technical efficiencies and technology gaps for firms operating under different technologies. J. Prod. Anal..

[20] O’Donnell C.J., Rao D.P., Battese G.E. (2008). Metafrontier frameworks for the study of firm-level efficiencies and technology ratios. Empir. Eco..

[21] Tiedemann T., Francksen T., Latacz-Lohmann U. (2011). Assessing the performance of German Bundesliga, football players: A nonparametric metafrontier approach. Central Eur. J. Oper. Res..

[22] Huang C.W., Ting C.T., Lin C.H., Lin C.T. (2013). Measuring nonconvex metafrontier efficiency in international tourist hotels. J. Oper. Res. Soc..

[23] Afsharian M. (2017). Metafrontier efficiency analysis with convex and nonconvex metatechnologies by stochastic nonparametric envelopment of data. Eco. Lett..

[24] Afsharian M., Podinovski V.V. (2018). A linear programming approach to efficiency evaluation in nonconvex metatechnologies. Eur. J. Oper. Res..

[25] Andersen P., Petersen N.C. (1993). A procedure for ranking efficient units in data envelopment analysis. Manag. Sci..

[26] Tone K. (2002). A slacks-based measure of super-efficiency in data envelopment analysis. Eur. J. Oper. Res..

[27] Swann G.M.P. (1996). Technology evolution and the rise and fall of industrial clusters. Rev. Int. Syst..

[28] Glaeser E.L., Kallal H.D., Scheinkman J.A., Shleifer A. (1992). Growth in cities. J. Political Eco..

[29] Melo P.C., Graham D.J., Noland R.B. (2009). A meta-analysis of estimates of urban agglomeration economies. Reg. Sci. Urban Eco..

[30] Cerina F., Mureddu F. (2014). Is agglomeration really good for growth? Global efficiency, interregional equity and uneven growth. J. Urban Eco..

[31] Márquez-Ramos L. (2015). The relationship between trade and sustainable transport: A quantitative assessment with indicators of the importance of environmental performance and agglomeration externalities. Ecol. Indic..

[32] Hu C., Xu Z., Yashiro N. (2015). Agglomeration and productivity in China: Firm level evidence. China Eco. Rev..

[33] Zheng Q., Lin B. (2018). Impact of industrial agglomeration on energy efficiency in China’s paper industry. J. Clean. Prod..

[34] Han F., Xie R., Fang J. (2018). Urban agglomeration economics and industrial energy efficiency. Energy.

[35] Hu A.H., Shih S.H., Hsu C.W., Tseng C.H. Eco-efficiency Evaluation of the Eco-industrial Cluster. Proceedings of the Fourth International Symposium on Environmentally Conscious Design and Inverse Manufacturing.

[36] Li W., Xu Y. (2013). Manufacturing agglomeration, environmental technological efficiency and energy-saving and emission-reduction. Eco. Manag. J..

[37] Shen N. (2014). Can industrial agglomeration improve environmental efficiency? —Spatial empirical test based on city data in China. J. Ind. Eng. Eng. Manag..

[38] Liu J., Cheng Z., Zhang H. (2017). Does industrial agglomeration promote the increase of energy efficiency in China?. J. Clean. Prod..

[39] Elhorst J.P. (2014). Spatial econometrics: From cross-sectional data to spatial panels. Springer Briefs in Regional Science.

[40] Ke S., Xiang J. (2012). Estimation of the fixed capital stocks in Chinese Cities for 1996–2009. Stat. Res..

[41] Arrow K. (1962). The economic implications of learning by doing. Rev. Eco. Stud..

[42] Marshall A. (1920). Principles of Economics.

[43] Romer P.M. (1990). Endogenous technological change. J. Political Eco..

[44] Jacobs J. (1969). The Economy of Cities.

[45] Porter M.E. (1990). The Competitive Advantage of Nations.

[46] Stern P.C., Young O.R., Druckman D. (1992). Global Environmental Change: Understanding the Human Dimensions.

[47] Dietz T., Rosa E.A. (1994). Rethinking the environmental impacts of population, affluence and technology. Hum. Ecol. Rev..

[48] Ren S., Li X., Yuan B., Li D., Chen X. (2018). The effects of three types of environmental regulation on eco-efficiency: A cross-region analysis in China. J. Clean. Prod..

[49] Javorcik B.S., Wei S.J. (2001). Pollution havens and foreign direct investment: Dirty secret or popular myth?. Contrib. Eco. Anal. Policy.

[50] Liu Q., Wang S., Zhang W., Zhan D., Li J. (2018). Does foreign direct investment affect environmental pollution in China’s cities? A spatial econometric perspective. Sci. Total Environ..

[51] Wang D.T., Chen W.Y. (2014). Foreign direct investment, institutional development, and environmental externalities: Evidence from China. J. Environ. Manag..

[52] Tone K. (2010). An epsilon-based measure of efficiency in DEA—A third pole of technical efficiency. Eur. J. Oper. Res..

[53] Simar L., Wilson P.W. (2007). Estimation and inference in two-stage, semi-parametric models of production process. J. Eco..

